# Optimization of the tracking beam sequence in Harmonic Motion Imaging

**DOI:** 10.1109/TUFFC.2023.3329729

**Published:** 2024-01-09

**Authors:** Yangpei Liu, Niloufar Saharkhiz, Md Murad Hossain, Elisa E. Konofagou

**Affiliations:** Department of Biomedical Engineering, Columbia University, New York, NY 10027 USA; Department of Biomedical Engineering, Columbia University, New York, NY 10027 USA; Department of Biomedical Engineering, Columbia University, New York, NY 10027 USA; Department of Biomedical Engineering and Radiology, Columbia University, New York, NY 10027 USA

**Keywords:** Breast cancer, Harmonic Motion Imaging, parallel tracking, simulation, speckle tracking, ultrasound elastography

## Abstract

Harmonic Motion Imaging (HMI) is an ultrasound elastography technique that estimates the viscoelastic properties of tissues by inducing localized oscillatory motion using focused ultrasound. The resulting displacement, assumed to be inversely proportional to the tissue local stiffness, is estimated using an imaging array based on RF speckle tracking. In conventional HMI, this is accomplished with plane wave (PW) imaging, which inherently suffers from low lateral resolution. Coherent PW compounding (PWC) leverages spatial and temporal resolution using synthetic focusing in transmit. In this study, we introduced focused imaging with parallel tracking in HMI and compared parallel tracking of various transmit F-numbers (F/2.6, 3, 4, 5) qualitatively and quantitatively with PW and PWC imaging at various compounded angle ranges (6°, 12°, 18°). An *in silico* model of a 56-kPa spherical inclusion (diameter: 3.6 mm) embedded in a 5.3-kPa background and a 5.3-kPa elastic phantom with cylindrical inclusions (Young’s moduli: 22 to 56 kPa, diameters: 2.0 to mm) were imaged to assess different tracking beam sequences. Speckle biasing in displacement estimation associated with parallel tracking was also investigated and concluded to be negligible in HMI. Parallel tracking in receive resulted in 2–7% and 8–12% increase compared to PW imaging (p<0.05) in HMI contrast and contrast-to-noise ratio *in silico* and phantoms. Focused imaging with parallel tracking in receive was concluded to be most robust among PW and PWC imaging for displacement estimation, and its preclinical feasibility was demonstrated in post-surgical human cancerous breast tissue specimens and *in vivo* murine models of breast cancer.

## I. Introduction

CHANGES in tissue mechanical properties are associated with the progression of pathologies, considering that diseases alter the high-level organization of flexible proteins in the extracellular matrix [1], [2], such as collagen fibers [3]–[5]. Ultrasound elastography [6], [7] estimates tissue mechanical properties and has been widely applied in disease diagnosis in the liver [8], [9], pancreas [10], breast [11], [12], prostate [13], [14], skeletal muscle [15] and thyroid [16]. A majority of these techniques use acoustic radiation force (ARF) [17] to remotely deform the tissue. By analyzing the ARF-induced mechanical perturbation, tissue mechanical properties are assessed [18].

The volume encompassed by the ARF beam path is referred to as the region of excitation (ROE), i.e., on-axis. Shear waves always emanate from the ROE and propagate outwards, i.e., off-axis. After measuring the off-axis tissue displacements, shear wave speeds can be estimated using the time of flight [19] and converted to shear moduli under the assumption of linearity, isotropy, and elasticity [20]. Therefore, shear wave-based methods (e.g., Shear Wave Elasticity Imaging (SWEI) [21]–[23], Comb-Push Ultrasound Shear Elastography (CUSE) [24], and Supersonic Shear Imaging (SSI) [25]) provide quantitative measurement of tissue stiffness. However, the commonly used time-of-flight algorithm averages the shear wave propagation over a lateral window of several millimeters, limiting the spatial resolution of the elastograms and becoming susceptible to distortion of shear waves [26]. In addition, shear wave induction and/or measurement can be challenging for patients with obesity [27] due to attenuation. Another group of ARF-based methods (e.g., Acoustic Radiation Force Impulse (ARFI) imaging [28], [29], Viscoelastic Response (VisR) ultrasound imaging [30], and Harmonic Motion Imaging (HMI) [31], [32]) measures the on-axis tissue displacement to assess its mechanical properties. Since the radiation force *in situ* is generally unknown, displacements per se can only provide qualitative estimates of mechanical properties. Nonetheless, displacement-derived images have been shown to provide informative diagnoses in tumor characterization [33]–[37]. Unlike shear wave-based methods, displacement-based methods are less prone to shear wave reflections and distortions [38].

In HMI [31], [39], a focused ultrasound (FUS) transducer is driven by an arbitrary function generator with a continuous amplitude-modulated (AM) pulse sequence to induce oscillatory tissue displacements. An imaging array, controlled by an ultrasound data acquisition (DAQ) system, is aligned confocally with the FUS transducer to estimate the resulting micron-level, localized, dynamic displacements within the FUS focal spot. In general, the estimated displacements are lower in stiffer tissues compared to softer tissues. Compared with ARFI imaging, the utilization of AM-ARF renders HMI more robust from various in-plane motion artifacts from the patients [40]. Benefiting from the known AM frequency, respiratory frequencies and others can be easily removed. Previously, HMI has been successfully applied in murine abdominal organ mapping [41], pancreatic [35], [42] and breast [34], [36], [37] cancer characterization, tumor response assessment to chemotherapy [35], and high intensity focused ultrasound (HIFU) ablation monitoring [43]–[48].

Plane wave (PW) imaging was primarily used in HMI to capture dynamic tissue displacements in an area of 2–40 mm^2^ at a frame rate of 1–2 kHz [34], [49]. However, PW imaging inherently suffers from low lateral resolution and contrast due to the lack of focus during transmission (Tx) [50]. Coherent plane wave compounding (PWC) [50]–[52] can mitigate this limitation by generating a posteriori synthetic focus in transmission at the cost of reducing the frame rate by a factor of the number of angles in compounding. Another imaging technique leveraging the lateral resolution and frame rate is parallel tracking in receive (Rx) with a focused transmit, here termed Rx parallel tracking [53]. Rx parallel tracking suffers from speckle biasing [26], [54] due to the inhomogeneous distribution of transmitted ultrasound energy across lateral lines to be parallelly beamformed. PW and PWC imaging, on the other hand, do not suffer from speckle biasing as they interrogate the field of view uniformly.

Given the aforementioned trade-offs, the objective of this study is to investigate the effect of different tracking beam sequences on the HMI image quality in terms of contrast and contrast-to-noise ratio (CNR). The tracking beam sequences to be examined cover plane wave imaging, coherently compounded plane wave imaging, and focused imaging with parallel tracking. An *in silico* model of homogeneous background with a spherical inclusion (n = 1) and a tissue-mimicking phantom with multiple cylindrical inclusions (n=7) were imaged. Lastly, preliminary feasibility of the optimized beam sequence are shown in *ex vivo* human cancerous breast tissue (IDC) and *in vivo* murine models of breast cancer (4T1).

## II. Materials and Methods

### A. HMI system

**Fig. 1:**
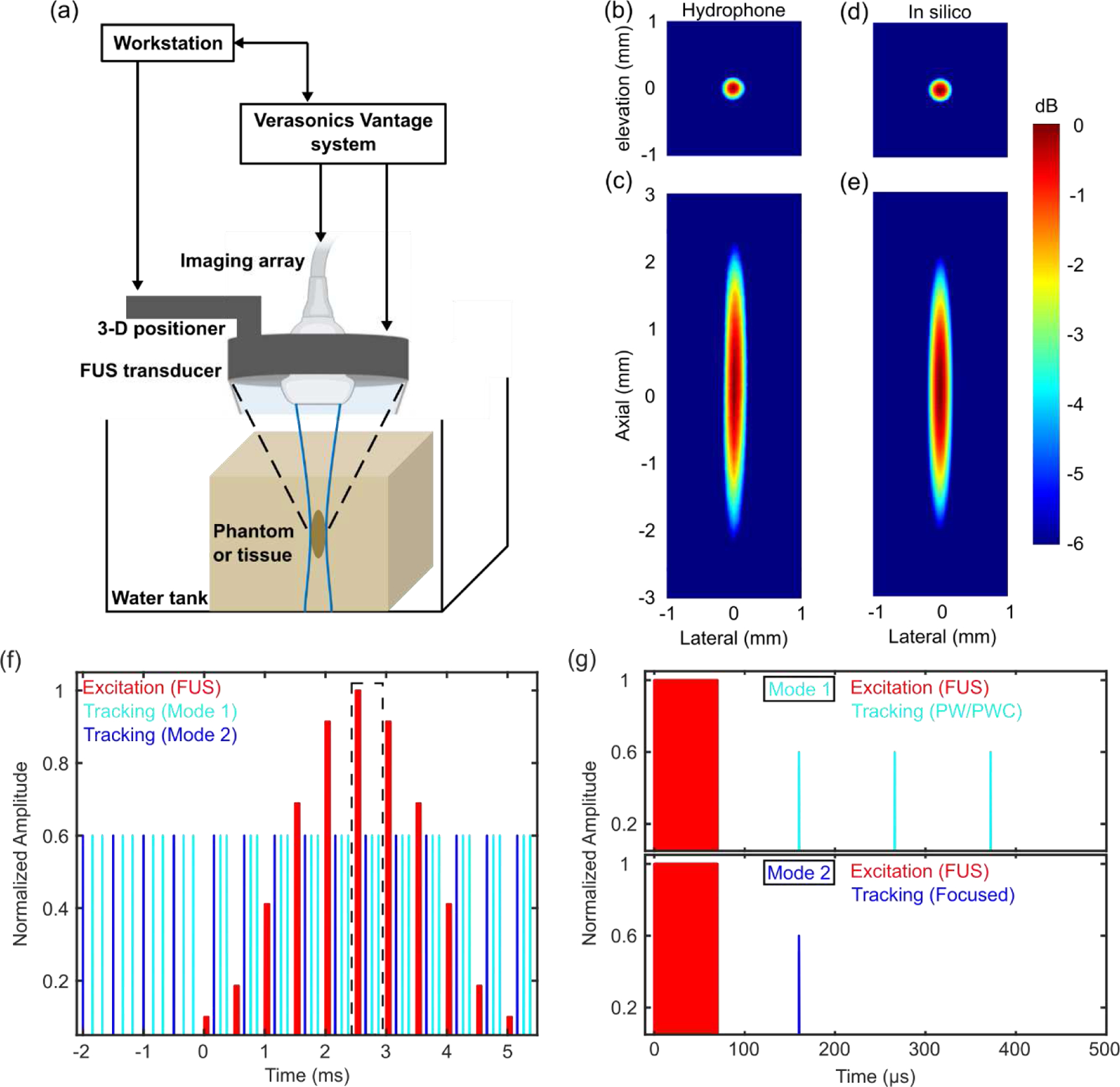
Schematic of the Harmonic Motion Imaging (HMI) setup, FUS transducer beam profiles measured in a water tank and simulated in Field-II, and interleaved HMI pulse sequence. (a) A 256-channel Vantage system controlling both the 128-element FUS transducer and the 104-element imaging array. The FUS focal volume and the imaging plane were co-aligned. The 2D HMI raster scan was controlled by a 3D positioner. Normalized pressure profiles of the FUS beam measured with a fiber-optic hydrophone and simulated in Field-II in the (b, d) transverse and (c, e) axial plane. (f) Pulsed 200-Hz amplitude-modulated excitation sequence (red) interleaved with tracking pulses (cyan if in the PW mode or blue if in the focused mode). The FUS pulse repetition frequency and imaging frame rate were 2 kHz (500 μs pulse interval). (g) Magnification of the pulse delineated with dashed black lines in (f) comprising a 70-μs FUS excitation pulse followed by tracking pulses. The time delay (90 μs) between excitation and tracking was optimized to remove most of the FUS interference on the channel signal received by the imaging array.

The single-system HMI [49] setup, shown in Fig. 1a, consisted of a 128-element FUS transducer (f_c_: 4.5 MHz, diameter: 80 mm, radius of curvature: 76 mm, H265, Sonic Concepts Inc., Bothell, WA, USA) with a custom RF matching network and a 104-element phased imaging array (f_c_: 7.8 MHz, P12–5, ATL Phillips, Bothell, WA, USA) confocally aligned with the FUS transducer using a custom 3D-printed attachment through the 41-mm central opening in the FUS transducer [55]. The free-field FUS pressure profile at the geometric focus (Fig. 1b, c) was measured in a tank filled with deionized and degassed water using a fiber-optic hydrophone (HFO-690, Onda Corp, Sunnyvale, CA, USA). The FUS focal spot (transverse x axial) was 0.39 mm x 4.3 mm. A 256-channel ultrasound research system (Vantage with HIFU options, Verasonics Inc., Kirkland, WA, USA) was used to control and synchronize the transducer assembly in an interleaved fashion. A 3D positioning system (Velmex Inc., Bloomfield, NY, USA) was used to translate the two transducers in a 2D point-by-point raster scanning format to obtain a 2D image [49]. The step sizes of the mechanical movement were 0.8 mm laterally and 2.0 mm axially optimized based on the dimensions of the FUS focal spot and the resulting displacement field to provide overlap between consecutive raster-scan points. The mechanical raster scanning range was tailored based on the lesion dimension identified from a pre-acquired B-mode image to cover sufficient background regions for post-processing.

### B. Interleaved HMI pulse sequence

The amplitude-modulated FUS excitation pulses were interleaved with the imaging tracking pulses. The sinusoidal amplitude modulation of excitation was realized by varying the relative pulse width [49], which generated oscillatory displacements for 30 ms, i.e., 6 cycles of 200-Hz AM. The optimal AM frequency to implement depends on the lesion dimension and stiffness [56], covering 200, 300, 600 and 800 Hz in this study. One exemplary cycle of interleaved HMI pulse sequence of 200-Hz AM is illustrated in Fig. 1f consisting of pulsed AM FUS excitation and interleaved tracking pulses. As shown in Fig. 1g, each pulse is 500 μs long, including a 70-μs FUS excitation followed by a time window of 430 μs for tracking pulses, generating a frame rate of 2 kHz for displacement estimation. Since the beams of the FUS and imaging were co-registered and there was bandwidth overlap between their operating frequencies, to avoid extensive FUS interference on the received channel signal by the imaging array, an idle time of 90 μs after each excitation pulse was implemented before the next tracking pulse started. When a 5-angle PWC sequence was implemented for tracking, this idle time was reduced to 40 μs to allow sufficient time for 5 individual PWs.

The co-aligned imaging array was programmed to work in synchrony with the FUS transducer and was able to operate either in the PW or focused mode. In both cases, an area (axial x lateral) of 3.2 mm x 1.2 mm around the FUS focus was reconstructed and used for displacement estimation. Since there was no limitation of the time window for transmitting tracking pulses *in silico*, a maximum number of 7 angles was implemented in PWC *in silico*; however, a maximum number of 5 angles could be achieved in phantoms. Tracking beam sequences of PW imaging, PWC imaging of 3, 5, and 7 angles (3° difference between consecutive angles), and focused imaging (Tx F/2.6, 3, 4, and 5) with Rx parallel tracking were compared. The entire aperture was always used for beamforming in receive, giving an Rx F/# of 2.6. When not stated otherwise, parallel tracking was always implemented in focused transmits.

**Fig. 2:**
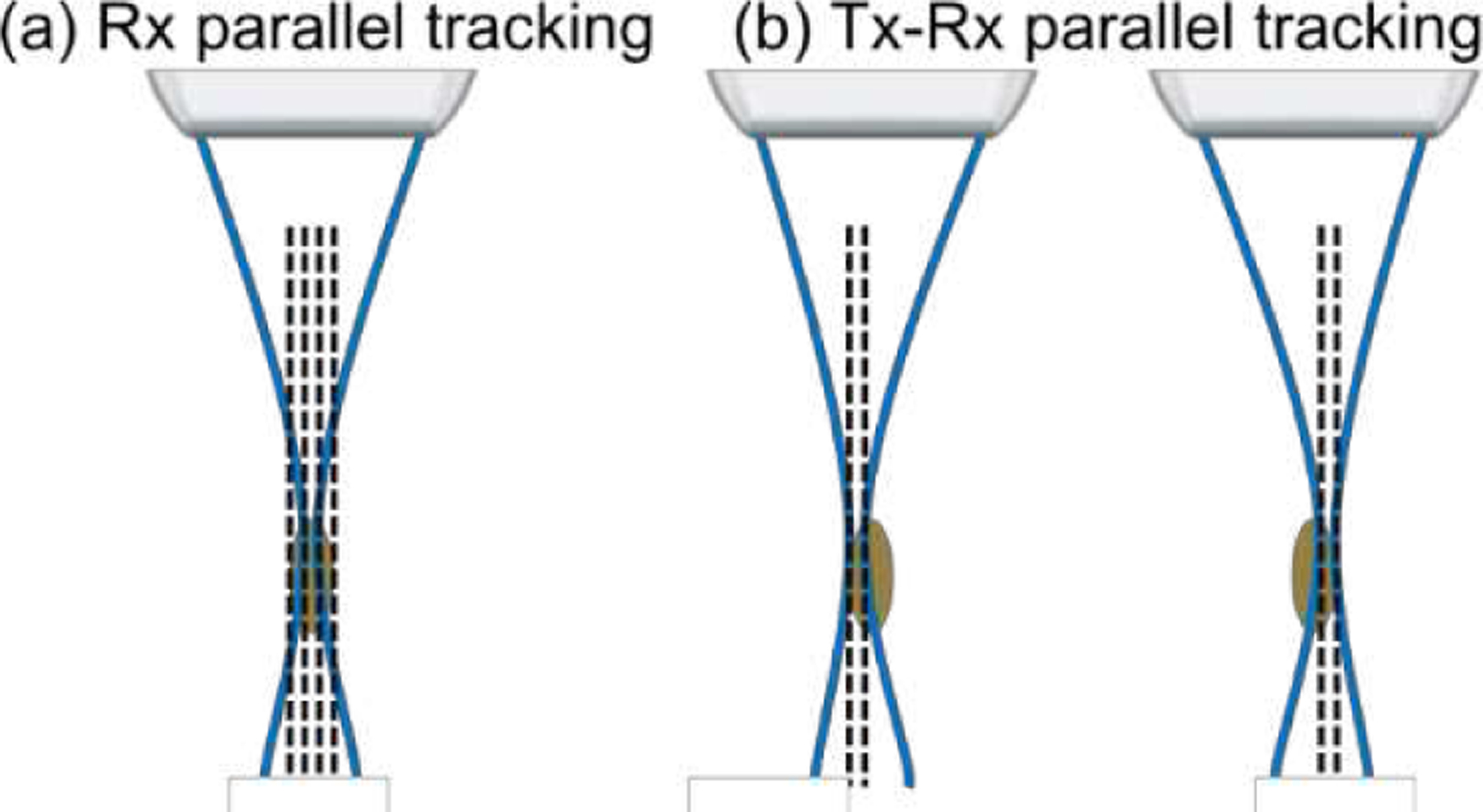
Schematic of different parallel tracking beam sequences co-aligned with the FUS focal spot (brown ellipse). Blue lines indicate the imaging beam path, and dashed black lines indicate image lines to be beamformed. (a) Conventional Rx parallel tracking. (b) Proposed Tx-Rx parallel tracking with two transmit-receive events offset laterally to cover the entire FUS focal spot.

Displacement estimation with Rx parallel tracking can be compromised due to biasing from the speckle [26], or positional error, as referred to in [54]. This happens when a tight transmit focus is used, and an inhomogeneous distribution of transmitted ultrasound energy is formed (Fig. 2a). Parallel beamforming of lines that are farther away from the center of the transmitted beam is unavoidably influenced by the backscattered signals from the center lines. As a result, displacement estimation can be biased towards the tissue motion surrounding the focus of the transmitted beam. To examine the effect of speckle biasing in HMI with Rx parallel tracking, cylindrical inclusions were imaged with the lateral raster scanning step size halved from 0.8 mm to 0.4 mm, suggesting fewer lines to be parallelly beamformed in receive and slower data acquisition speed. To maintain the same data acquisition speed, a novel tracking beam sequence, termed Tx-Rx parallel tracking in Fig. 2b, was implemented, where two transmit-receive events offset laterally (0.4 mm apart, half of the lateral raster scanning step size) were fired sequentially after each excitation to cover the entire reconstruction area. The received channel data were then beamformed independently and combined in a way similar to focused imaging with multiple focal zones. However, this combination was done laterally, unlike in dynamically focused imaging, which combines axially. One can also maintain the original raster scanning step size and interpolate the lines further away from the center of transmit beams during post-processing. Lastly, by transmitting a broadly focused beam with a large F/#, such as F/5, speckle biasing could be partially reduced with a more balanced energy distribution in transmission across lines to be parallelly beamformed. HMI image quality in terms of contrast and CNR was compared between all the above-mentioned sequences. Displacement SNR was also reported for simulations.

### C. In silico model

The *in silico* model was adapted from [57], which was based on Field II [58], [59] LS-DYNA3D (Livermore Software Technology Corp, Livermore, CA, USA). The modified model in this study was able to perform both PW and focused tracking with Rx parallel tracking of FUS-induced displacements. The simulated FUS pressure profile at the geometric focus in Field II is shown in Fig. 1d, e, in good agreement with the hydrophone measurement. The same imaging array (P12–5) as used in the phantom experiments was simulated in Field II for speckle tracking at a sampling frequency of 1 GHz. The FEM mesh consisted of a 5.3-kPa (Young’s modulus) spherical inclusion (diameter: 3.6 mm) embedded in a 56-kPa background, with Young’s viscosity of 0.6 Pa·S [21] for both the background and inclusion. The element size of the FEM mesh was 0.1 mm [30], [37] in each direction to adequately sample the FUS focal spot. The simulated FUS pressure field was converted to the intensity field and applied to the FEM model to solve for the particle displacement. Half symmetry along the elevation direction was used in the FEM model to reduce computational overhead. 2D point-by-point raster scans of a two-cycle HMI sequence of 200-Hz AM were simulated. Twenty independent speckle realizations with 20 scatterers per resolution cell in Field II were displaced according to the FEM displacement estimates at a frame rate of 2 kHz. 30-dB band-limited Gaussian noise was added to the simulated channel signal prior to beamforming [60] to match the Vantage system channel signal-to-noise ratio (SNR). Rx parallel tracking (Tx F/2.6, 3, 4, 5), PW imaging, and PWC imaging of 3, 5, and 7 angles were simulated and compared.

### D. Phantom experiments

To compare the performance of Rx parallel tracking with PW and PWC imaging in HMI, nine stepped-cylindrical inclusions with properties mimicking human breast tumors [12], [61] in a custom elastic phantom (background Young’s modulus: 5.3 kPa, CIRS, Norfolk, VA, USA) were imaged. In experiment 1, inclusions (diameter, Young’s modulus): (2.6, 4.6, 7.4 mm, 22 kPa), (3.0, 5.0, 7.8 mm, 31 kPa), (3.2, 5.8, 8.6 mm, 56 kPa), were scanned with an AM frequency of 200 Hz. The data acquisition time for (lateral x axial) small (9.2 × 11.2 mm), medium (10.8 × 15.2 mm), and large (15.6 × 19.2 mm) fields of view were around 79, 132, and 250 seconds for Rx parallel tracking or PW imaging, 82, 138, and 261 seconds for 3-angle PWC, and 86, 145, and 271 seconds for 5-angle PWC, respectively.

Since no detectable HMI image quality difference could be found in experiment 1 between Rx parallel tracking with different Tx F/#s, another three inclusions were imaged in experiment 2 to investigate the effect of speckle biasing associated with Rx parallel tracking in HMI: (2.0 mm, 56 kPa), (2.8 mm, 56 kPa), and (3.2 mm, 56 kPa) with higher AM frequencies (300, 600, 800 Hz) [56]. The reported inclusion diameters were measured in the B-mode images acquired before HMI, and Young’s moduli were provided by the manufacturer. 2D point-by-point raster scans of HMI were performed with the phantom immersed in a tank of deionized and degassed water. For each point scan, the 30-ms pulsed HMI sequence was used. Six independent random speckle realizations for each inclusion were imaged by moving the transducers in the elevation direction using the 3D positioning system. Rx parallel tracking (Tx F/2.6 and 5 with 0.8 mm lateral raster scanning step size, Tx F/2.6 with 0.4 mm lateral raster scanning step size), Tx-Rx parallel tracking (Tx F/2.6) were performed and compared. The interpolation method mentioned in section IIB was also tested with Rx parallel tracking (Tx F/2.6 with 0.8 mm lateral raster scanning step size).

### E. Ex vivo human breast experiment

The clinical feasibility of the optimized tracking beam sequence was demonstrated in an *ex vivo* human breast tumor specimen following the protocol approved by the institutional review board (IRB) of Columbia University (protocol#: AAAT4412). A 74-year-old female patient diagnosed with a 2.8-mm invasive ductal carcinoma (IDC) tumor was recruited with written consent. Clinical B-mode images acquired before the mastectomy were retrieved from the hospital. Breast specimens were collected and imaged in the lab within 1 hour of mastectomy. The *ex vivo* specimen (n=1) was immersed in phosphate-buffered saline during the imaging session. 2D HMI raster scans of 200-Hz AM with the same parameters as used in the phantom experiments were performed.

### F. In vivo murine model of breast cancer experiment

The animal protocol was reviewed and approved by the Columbia University Irving Medical Center (CUIMC) Institutional Animal Care and Use Committee (IACUC). A 4T1 (triple-negative breast cancer) orthotopic murine model [62] was used to validate the *in vivo* performance of the optimized tracking beam sequence for HMI. 8-week-old immune-compromised BALB/c mice (n = 2, Jackson Laboratory) were inoculated with $5\times 10^{4}$ 4T1 cells/mouse in the fourth mammary fat pad one day after shaving. The mouse tumors were imaged in the supine position 5 weeks after inoculation. During the imaging session, the mice were anesthetized with 1–2% isoflurane. Respiratory gating [41] was applied during the 2D HMI raster scan of 200-Hz AM. A thin layer of ultrasound gel was applied between the transducers and the mouse mammary fat pad to provide acoustic coupling.

### G. HMI channel data post-processing

Channel data from the Vantage research system was sampled at 4x the center frequency of the imaging array and transferred to a local PC. HMI displacement estimation was done offline in MATLAB (R2019b, MathWorks, Natick, MA, USA). A GPU-accelerated delay-and-sum algorithm [44] was implemented to reconstruct the stored channel signal to beamformed RF lines with an upsampling factor of 4. Since there were overlaps between the operating frequency bands of the imaging array (5–12 MHz) and the fundamental, 2^nd^, and 3^rd^ harmonics of the FUS (4.5, 9, 13.5 MHz), a bank of second-order Butterworth notch filters was designed to suppress the FUS interference in the RF lines. Interframe axial displacements were estimated using 1D normalized cross-correlation (NCC) [63] with a window of 4λ (0.79 mm, λ being the wavelength of the imaging center frequency) and 95% overlap [64], [65]. These parameters were selected empirically to provide an adequate balance between processing speed and displacement estimation accuracy [49], [66]. Cosine interpolation was applied to improve the precision of the displacement estimation [67]. A 48^th^-order FIR band-pass filter was designed to extract the oscillatory HMI displacement at the applied AM frequency. Next, mean peak-to-peak (P2P) HMI displacement over cycles was estimated. Then, a P2P displacement image of 3.2 mm axially by 1.2 mm laterally around the FUS focus was produced at each scanning point. This field of view was slightly larger than the raster scanning step sizes using the 3D positioner, which was done on purpose to provide overlaps between raster scan points. The series of P2P displacement images were combined to form a 2D HMI displacement map with overlap blending using weights determined linearly by the pixel distance to the raster scan point with higher weights for pixels closer to the raster scan point. Acoustic radiation force attenuation over depth was compensated by normalizing the 2D HMI displacement map axially by a displacement curve averaged from a homogeneous background region identified from the B-mode image. Therefore, in the final HMI displacement map, the parameter displayed was “ratio” with respect to the background displacement and lower values indicate stiffer tissue. When no background tissue was available for attenuation correction at certain depths, e.g., 26–31 mm in Fig. 10, normalizing functions were extrapolated by fitting a Gaussian function [37].

Using a 3.1 GHz Intel Xeon CPU with 16 cores and 2 threads cores and 2 threads per core was 0.9867 (Rx parallel tracking or plane wave imaging), 1.1569 (3-angle PWC), and 1.4237 (5-angle PWC) seconds per raster scanning point. For a field of view of 15.2 mm laterally and 18 mm axially, 171 scanning points were required, which resulted in a post-processing time of 2.8121, 3.2972, and 4.0575 minutes, respectively. To optimize the execution time and achieve real-time post-processing, parallel processing using CUDA-optimized code [68] could be implemented.

### H. Image quality metrics and statistical analysis

The inclusion boundaries were derived from the B-mode images by one observer (YL) and used for HMI image quality evaluation. To quantitatively compare the performance of different tracking beam sequences in HMI, contrast and CNR were calculated using identically sized regions of interest (ROIs) in the inclusion and background. The inclusion ROIs, delineated with red contours in the following B-mode images, were defined as concentric circles with around 80% of the corresponding inclusion diameter. The background ROIs, delineated as green, were defined as concentric rings surrounding the inclusion with an inner diameter of ~170% of the corresponding inclusion diameter. Contrast, CNR, and SNR in the background or inclusion were defined as $\left|\mu_{b}\;-\;\mu_{i}\right|/\mu_{b}\;$, $\left|\mu_{b}\;-\;\mu_{i}\right|/\sqrt{\left(\sigma_{b}^{2}\;+\;\sigma_{i}^{2}\right)}$, and μ/σ. $\mu_{b}$, $\mu_{i}$, $\sigma_{b}$, and $\sigma_{i}$ are the median and standard deviation of the normalized HMI displacements in the background and inclusion. Since a 5–10% standard deviation in the nominal Young’s moduli was reported by the manufacturer for the elastic phantoms, displacement SNR was only reported *in silico*. Due to the irregular lesion shape in the *ex vivo* and *in vivo* biological tissues, contrast and CNR were calculated in rectangular ROIs covering an identical area at the same depth. The displacement ratio (DR) [56], defined by $\mu_{i}/\mu_{b}$, was also reported for biological tissues. Statistical analyses of contrast and CNR between different tracking beam sequences over 20 or 6 independent speckle realizations *in silico* or phantoms, respectively, were performed in Prism 7 (GraphPad Software, La Jolla, CA, USA). A Friedman test, a non-parametric ANOVA test, was first performed across all tracking beam sequences. If a statistically significant group difference was found, post-hoc Dunn’s rank-sum tests were performed to identify specific differences between the sequence with the highest median and the rest. A p-value less than 0.05 was defined as statistically significant throughout this study.

## III. Results

### A. Experiment 1: Rx parallel tracking in HMI

**Fig. 3:**
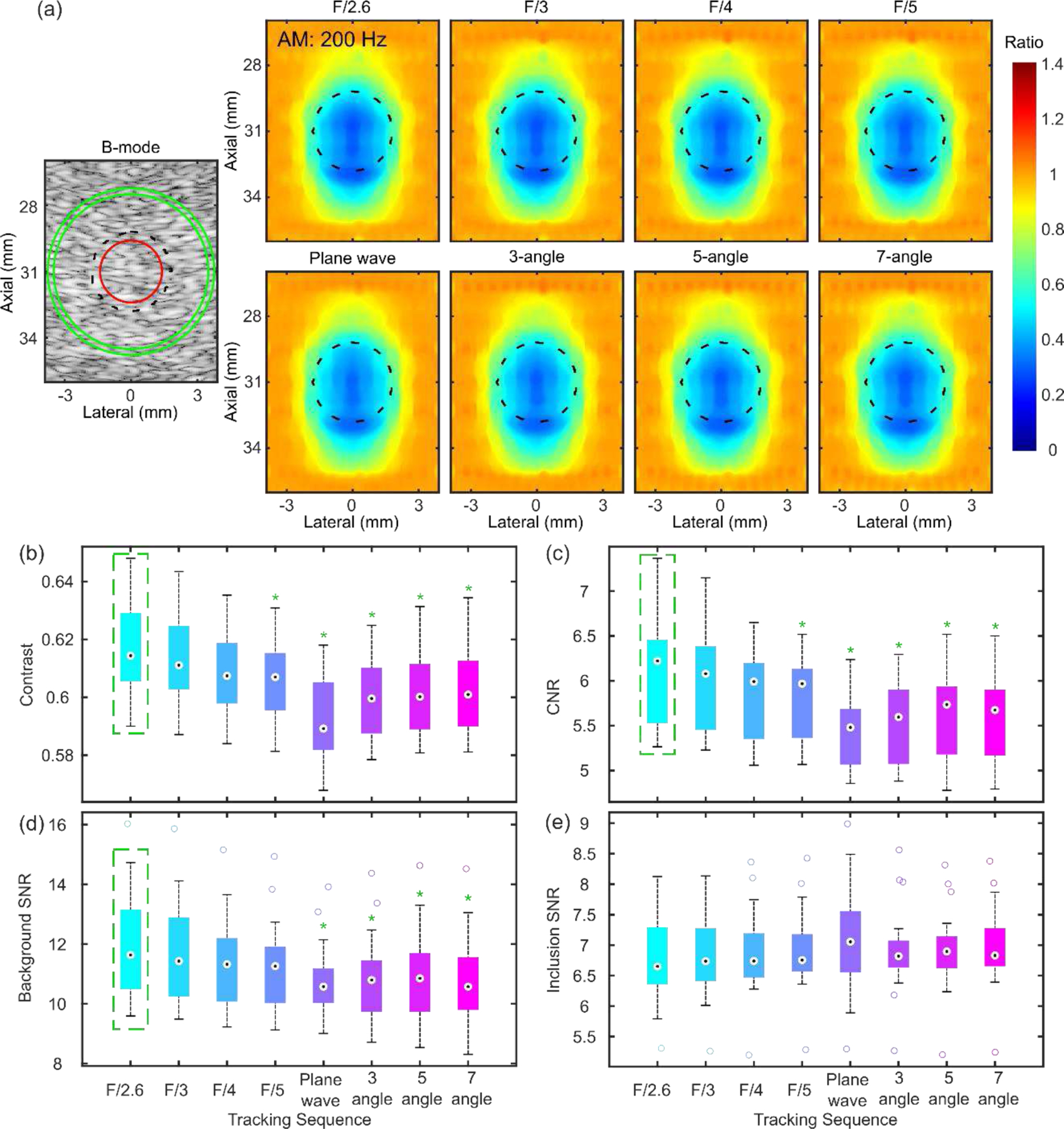
Summary of *in silico* results of a 56-kPa spherical inclusion (diameter: 3.6 mm) embedded in a 5.3-kPa background with HMI of 200-Hz AM. (a) B-mode, normalized HMI displacement maps obtained by Rx parallel tracking with various transmit F-numbers, PW, and PWC imaging with various compounding angles. Black, red, and green contours represent the inclusion boundary and ROIs for image quality evaluation in inclusion and background. Boxplots of (b) contrast, (c) CNR, and SNR in the (d) background and (e) inclusion of HMI images over 20 independent speckle realizations. Friedman test indicated that displacement contrast, CNR, and background SNR were statistically different across different tracking sequences. Post-hoc Dunn’s test was performed between the group with the highest median (dotted green rectangle) and the rest groups. A green asterisk (*) indicates statistically significant differences from Dunn’s test.

*In silico* results of an isoechoic 3.6-mm, 56-kPa inclusion embedded in a 5.3-kPa background are shown in Fig. 3. Fig. 3a shows representative B-mode and normalized HMI (200-Hz AM) displacement maps obtained by Rx parallel tracking with various transmit F-numbers (F/2.6, 3, 4, 5), PW, and PWC imaging with various compounding angles (3, 5, 7) from one speckle realization. All tracking beam sequences were able to detect the inclusion, with the Rx parallel tracking sequences constantly having higher apparent contrast than the PW sequence. There was almost no observable difference between images obtained by Rx parallel tracking and PWC imaging. Boxplots in Fig. 3b summarize contrast and CNR across these sequences over 20 independent speckle realizations. The whisker length in each boxplot denotes one interquartile range. The Friedman test indicated that contrast, CNR and background SNR were statistically different across sequences (p<0.05). The maximum contrast, CNR, background SNR were achieved with Rx parallel tracking with Tx F/2.6, about 4%, 12%, and 7% higher than PW imaging. There were overall 2.3% and 8.9% increase in contrast and CNR with the focused transmit of F/2.6 compared to PWC imaging in average. Notice that ultrasonically estimated inclusion displacement contrast from each Tx-Rx sequence was all lower than that was measured directly from the FEM results, 0.8, and the ground truth Young’s modulus contrast, 0.9.

**Fig. 4:**
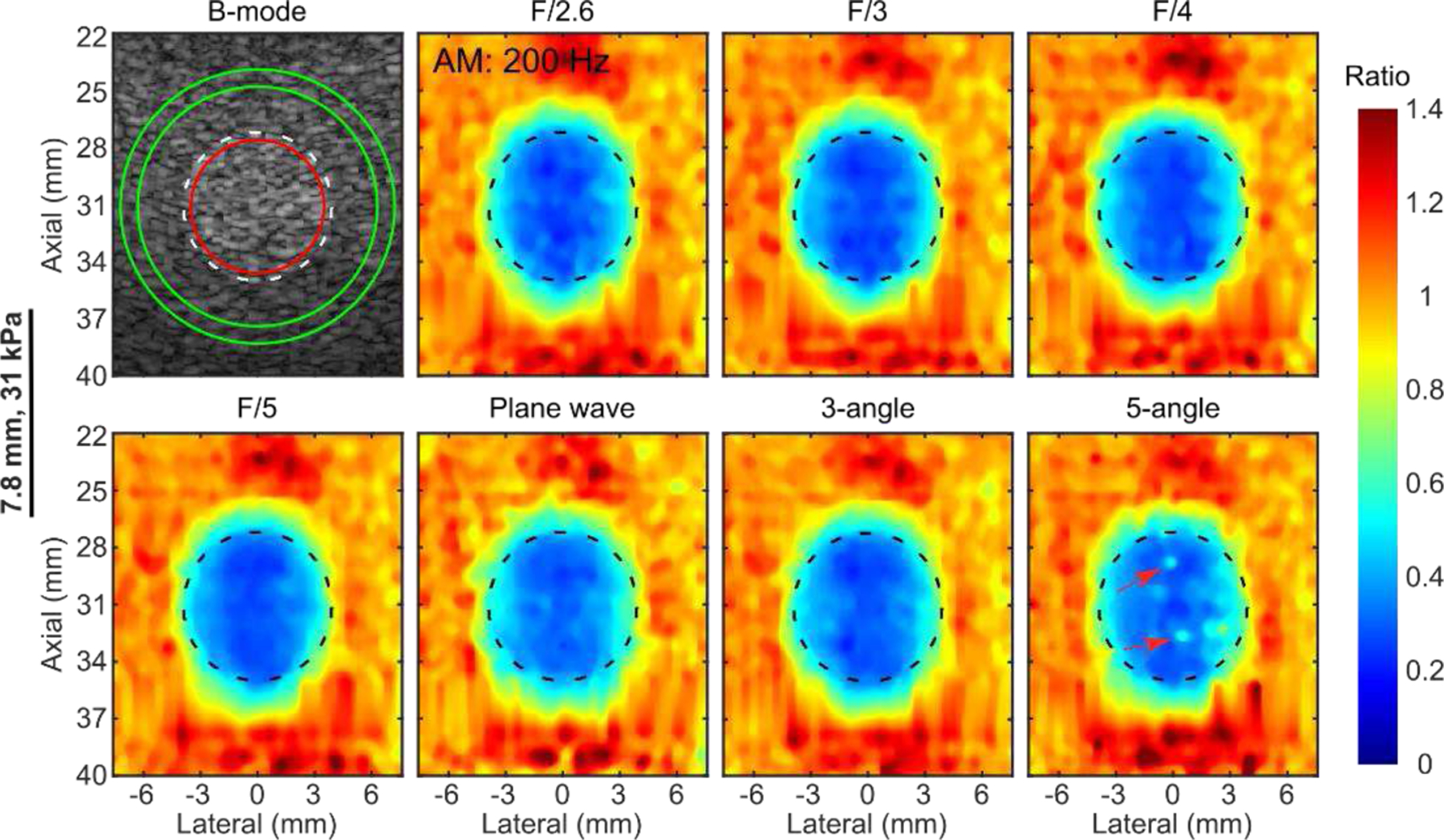
Representative B-mode and normalized HMI displacement maps of 200-Hz AM obtained by Rx parallel tracking with various transmit F-numbers, PW, and PWC imaging with various compounding angles of a 31-kPa cylindrical inclusion (diameter: 7.8 mm) embedded in a 5.3-kPa background. Dashed black or white and solid red and green contours represent the inclusion boundary derived from the B-mode and ROIs for image quality evaluation in inclusion and background. Red arrows in the HMI map obtained by 5-angle PWC indicate outliers from displacement estimation. The artifacts at around 39 mm depth in the HMI images may be due to the reverberations from the reflective plastic phantom membrane.

**Fig. 5:**
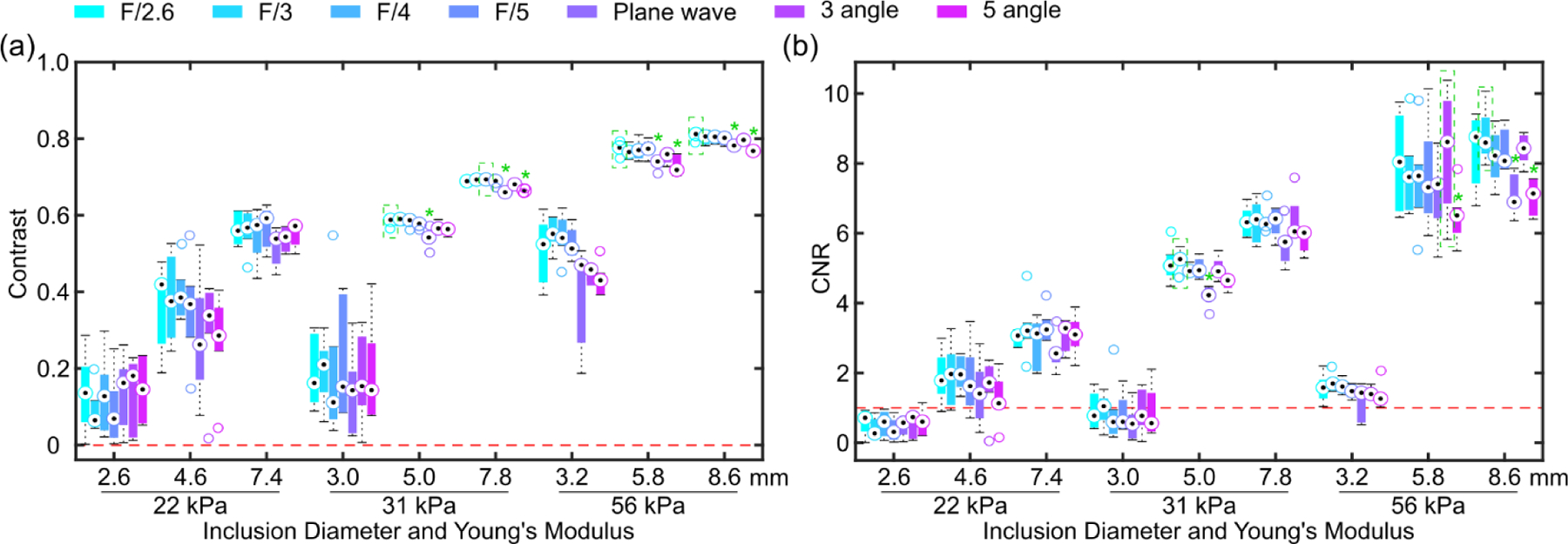
Summary of image quality of HMI displacement maps of 200-Hz AM obtained by Rx parallel tracking with various transmit F-numbers, PW, and PWC imaging with various compounding angles of cylindrical inclusions with various diameters and Young’s moduli embedded in a 5.3-kPa background. Boxplots of (a) contrast and (b) CNR of HMI images over 6 elevational positions for each inclusion. Friedman test was performed across different tracking sequences. Post-hoc Dunn’s test was performed between the group with the highest median (dotted green rectangle) and the rest groups if a statistically significant difference was found from the Friedman test. A green asterisk (*) indicates statistically significant differences from Dunn’s test.

Fig. 4 shows representative B-mode and normalized HMI (200-Hz AM) displacement maps obtained by Rx parallel tracking with various transmit F-numbers (F/2.6, 3, 4, 5), PW, and PWC imaging with various compounding angles (3, 5) of a 7.8-mm, 31-kPa inclusion embedded in a 5.3-kPa background. Focused transmits consistently produced higher perceived contrast than PW imaging, regardless of the Tx F/#. The 3-angle PWC sequence delineated the inclusion with contrast comparable to focused transmits. However, there were a few outliers (indicated by the red arrows) inside the inclusion in the HMI map obtained by the 5-angle PWC sequence. Fig. 5 summarizes contrast and CNR in phantoms across different tracking beam sequences. Notice that for small inclusions with a low Young’s modulus (2.6 mm, 22 kPa, and 3.0 mm, 31 kPa), 200-Hz AM was not able to resolve these inclusions with CNR less than 1. For inclusions with CNRs greater than 1, Rx parallel tracking had averaged 5.2% and 10% increase in contrast and CNR compared to PW imaging. However, there was no statistically significant difference between Rx parallel tracking with different transmit F-numbers and the 3-angle PWC sequence.

### B. Experiment 2: Tx-Rx parallel tracking in HMI

**Fig. 6:**
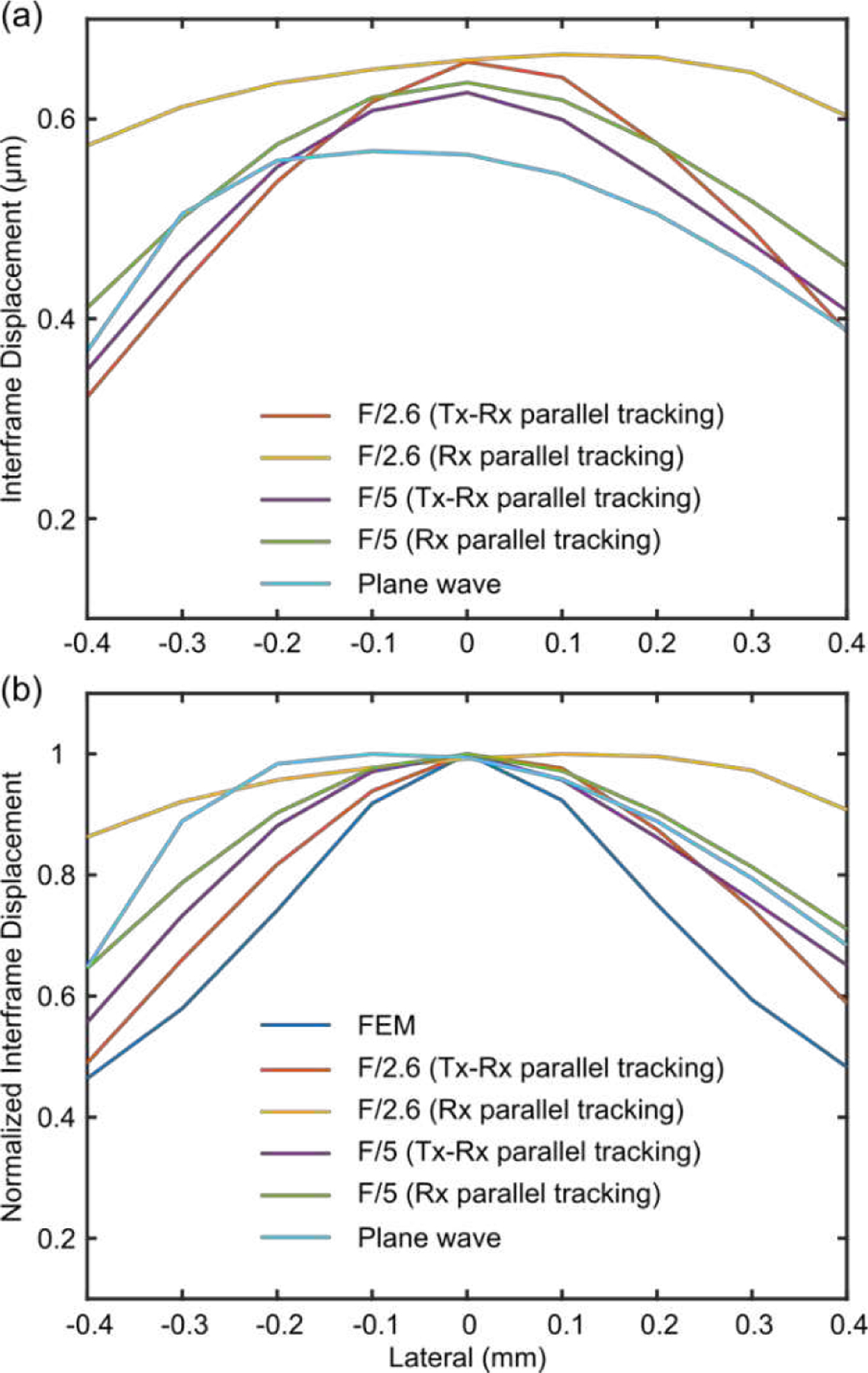
The effect of speckle biasing in displacement estimation associated with Rx parallel tracking with focused transmits *in silico*. (a) Lateral profiles of interframe particle displacements obtained by Rx parallel tracking with Tx F/2.6 and 5, Tx-Rx parallel tracking with Tx F/2.6 and 5, and PW imaging. (b) Lateral profiles of normalized interframe particle displacements compared with estimated displacements from the FEM model.

Fig. 6 illustrates the effect of speckle biasing in displacement estimation *in silico* due to the inhomogeneous lateral distribution of ultrasound energy in transmission. In Fig. 6b, the estimated displacement profiles from ultrasonic tracking appeared wider than the true displacement profile extracted directly from the FEM model due to shearing and speckle biasing. As expected, Rx parallel tracking with a tight focus (F/2.6) in transmission had the widest profile due to the strongest amount of speckle biasing. Tx-Rx parallel tracking with the same Tx F/2.6 produced the narrowest profile closest to the true displacement profile, indicating the capability of Tx-Rx parallel tracking to reduce speckle biasing. Interestingly, in Fig. 6a, displacement underestimation [29] was observed when a broad focus (F/5) was applied in transmission, with PW imaging having the highest amount of underestimation. Nevertheless, interframe displacements are usually normalized in HMI, so displacement underestimation per se was not a compromising factor in this study.

**Fig. 7:**
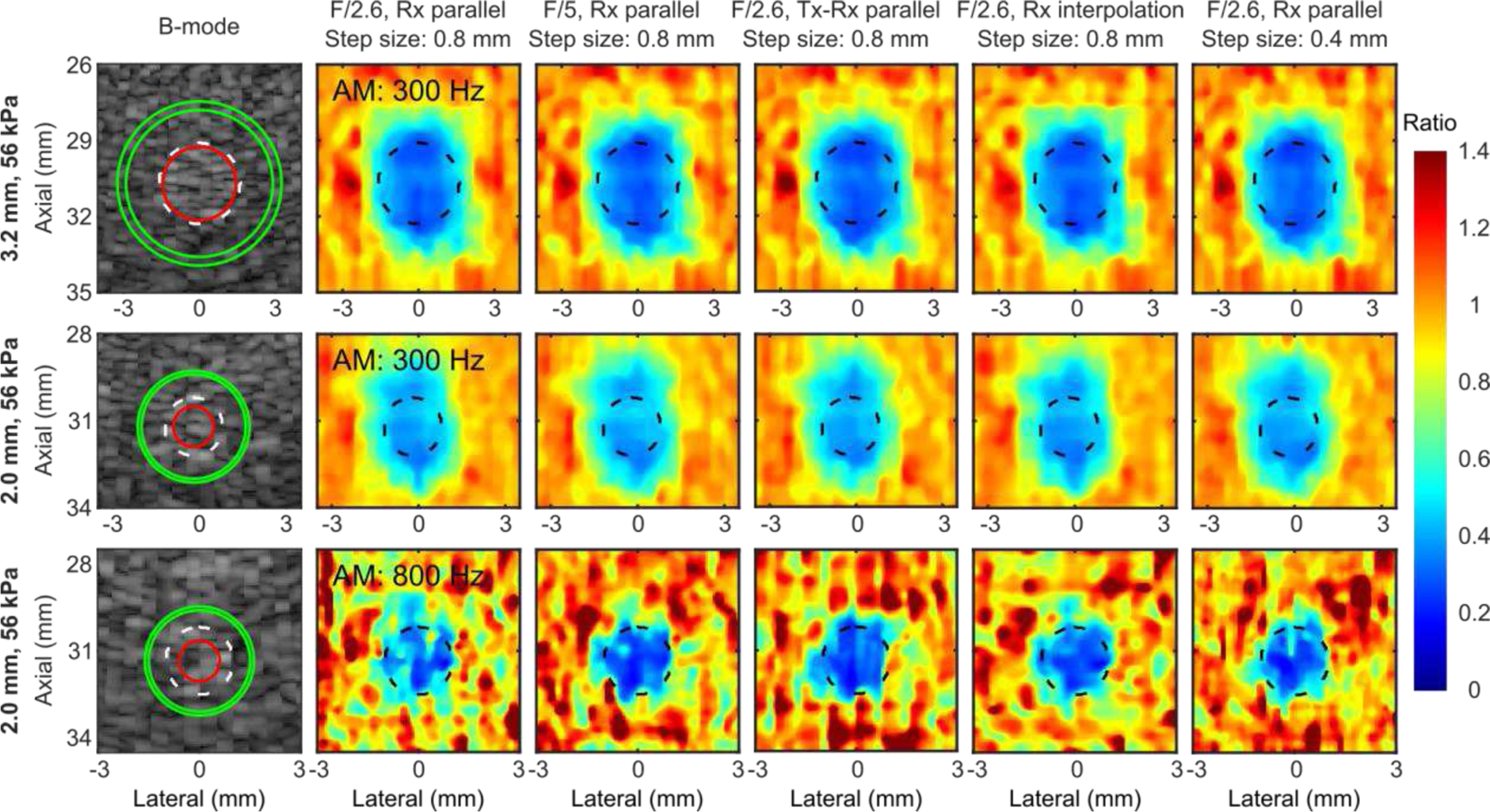
Representative B-mode and normalized HMI displacement maps obtained by parallel tracking with different transmit modes, lateral raster scanning step sizes, and image interpolation methods of 56-kPa inclusions embedded in a 5.3-kPa background. (1^st^ row) 3.2-mm inclusion with 300-Hz HMI. (2^nd^ row) 2.0-mm inclusion with 300-Hz HMI. (3^rd^ row) 2.0-mm inclusion with 800-Hz HMI. Dashed black or white and solid red and green contours represent the inclusion boundary derived from the B-mode and ROIs for image quality evaluation in inclusion and background.

**Fig. 8:**
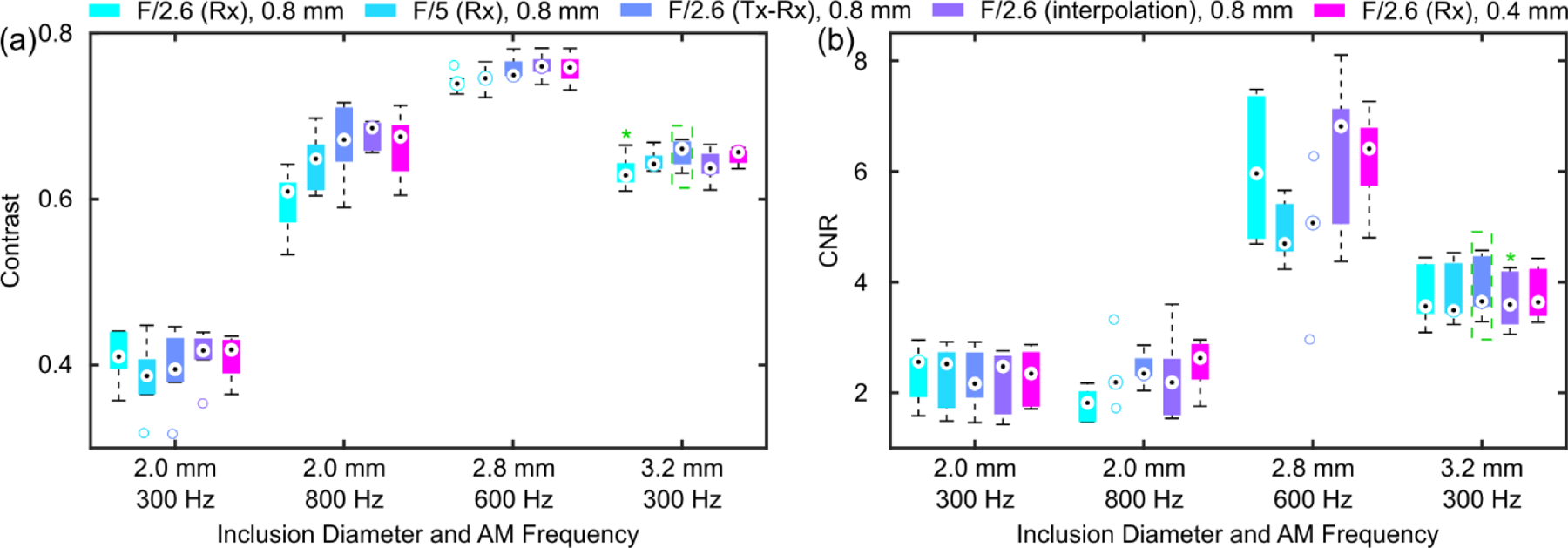
Summary of image quality of HMI displacement maps obtained by parallel tracking with different transmit modes, lateral raster scanning step sizes, and image interpolation methods of 56-kPa inclusions with various diameters embedded in a 5.3-kPa background and implemented AM frequency. Boxplots of (a) contrast and (b) CNR of HMI images over 6 independent speckle realizations for each inclusion and AM frequency combination. Friedman test was performed across different tracking sequences. Post-hoc Dunn’s test was performed between the group with the highest median (dotted green rectangle) and the rest groups if a statistically significant difference was found from the Friedman test. A green asterisk (*) indicates statistically significant differences from the Dunn’s test.

Fig. 7 shows representative B-mode and normalized HMI displacement maps obtained by parallel tracking with different transmit modes, lateral raster scanning step sizes, and image interpolation methods of 56-kPa inclusions with various diameters embedded in a 5.3-kPa background. There was no observable qualitative difference between different data acquisition sequences. For small inclusions (2.0 mm), the higher AM frequency (800 Hz) in HMI delineated the inclusion with a more well-defined boundary, closer to the derived boundary from the B-mode image, and higher perceived contrast compared to the lower AM frequency (300 Hz). Fig. 8 summarizes contrast and CNR in phantoms across different data acquisition sequences. There was no consistent statistical difference between different sequences. Although the higher AM frequency produced a higher contrast for the 2-mm inclusion, the contrast was similar between the two AM frequencies.

### C. Preclinical applications of Rx parallel tracking in HMI

**Fig. 9:**
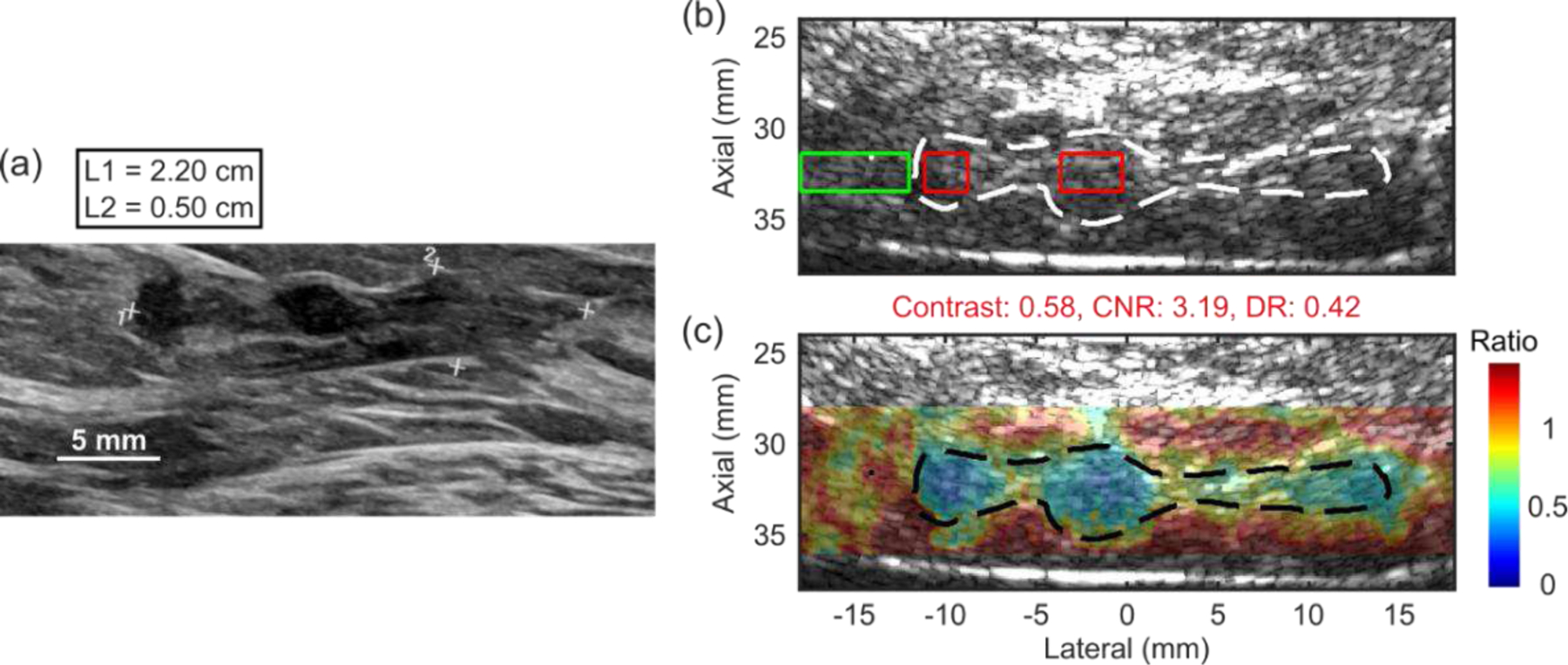
(a) Clinical and (b) research B-mode images from a patient diagnosed with invasive ductal carcinoma (IDC). (c) Normalized HMI displacement map overlaid on the research B-mode of the *ex vivo* human breast tumor. The tumor boundaries are delineated with dashed lines. The attenuation curve was estimated using the area to the left of the contoured tumor volume from around −18 to −13.5 mm laterally.

**Fig. 10:**
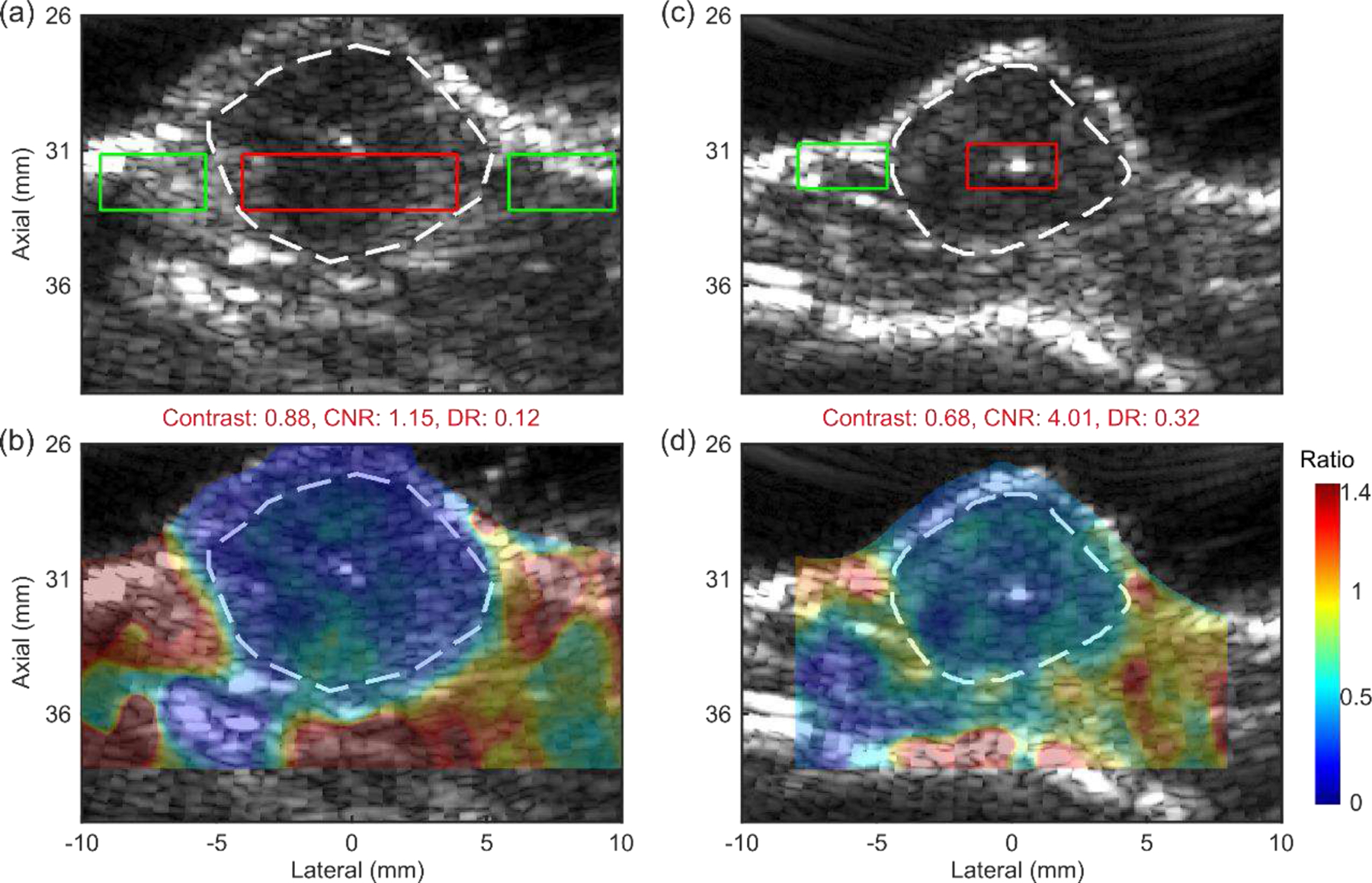
(a, c) B-mode images and (b, d) normalized HMI displacement maps overlaid on the B-mode of two *in vivo* murine breast tumors (4T1). The tumor boundaries are delineated with dashed lines.

Figs. 9 and 10 demonstrate the preclinical applications of Rx parallel tracking (Tx F/2.6) in HMI for breast tumor Characterization. Fig. 9 shows an *ex vivo* human cancerous breast tissue obtained from a female patient diagnosed with IDC. Tumor boundaries were drawn based on one observer on the clinical B-mode image. Fig. 10 shows two *in vivo* 4T1 murine tumors. Tumor boundaries were derived from the research B-mode acquired with the P12–5 imaging array. In the overlaid HMI images, the tumor boundaries and surrounding tissues were well-delineated and differentiated in both *ex vivo* human specimens and *in vivo* murine models with a contrast around or higher than 0.6.

## IV. Discussion

HMI [31], [39] is an oscillatory ARF-based elasticity imaging technique that measures the mechanical properties of the underlying tissue by inducing oscillatory tissue displacements using a FUS transducer. The resulting dynamic displacements are estimated using a 1D NCC-based motion estimation algorithm [63] based on RF speckle tracking by an imaging array, which is co-aligned with FUS. Since displacement tracking at high frame rates (> 500 Hz) is needed over a wide spatial region, conventional HMI uses PW imaging for speckle tracking to estimate tissue displacements within an area of 2–40 mm^2^, covering a lateral range of 1–3 mm. However, PW imaging inherently suffers from low spatial resolution and contrast [50]. This paper introduces Rx parallel tracking with focused transmits (Fig. 2a) in HMI to image quality in lesion characterization in terms of contrast and CNR. A comprehensive comparison between Rx parallel tracking with different transmit F/#, PW, and PWC imaging (experiment 1) was conducted *in silico* (Fig. 3) and tissue-mimicking phantoms (Figs. 4 and 5). The same experimental setup in phantom experiments was simulated in the FEM and Field II model. *In silico* findings were in good agreement with results from phantom experiments. Consistent statistically significant differences from the Dunn’s rank sum test (p<0.05) were seen between PW imaging and Rx parallel tracking in HMI contrast and CNR in both simulations and phantoms. Rx parallel tracking had overall 2–4% and 8–12% improvement in contrast and CNR compared to PW and PWC imaging *in silico*. Meanwhile, phantom experiments showed around 4–7% and 10% increase in contrast and CNR with Rx parallel tracking compared to PW imaging. However, the 3-angle PWC sequence had HMI image quality comparable to Rx parallel tracking in phantoms.

In simulations, ultrasonically estimated inclusion displacement contrast ranging from 0.56 to 0.65 (Fig. 3b) was slightly lower than the true inclusion displacement contrast of 0.8 measured directly in FEM. Three factors may partially explain this phenomenon. First, displacement underestimation due to the shearing effect [29] could unevenly affect the displacement estimation inside or outside the inclusion, potentially leading to a decrease in displacement contrast. Second, the definite imaging system point spread function works as a low-pass filter, which unavoidably produces an image contrast lower than the ground truth. Lastly and most importantly, as observed in Figs. 5 and 8, the HMI displacement contrast partially depends on the dimension of the inclusion and the applied AM frequency, which is consistent with the findings of our previous studies [56], [66]. That may explain why the HMI displacement contrast of 0.6 of the in silico 3.6-mm, 56-kPa inclusion was lower than 0.7 of the experimental 7.8-mm, 31-kPa inclusion (Fig. 5a).

The effect of speckle biasing in Rx parallel tracking due to inhomogeneous lateral distribution of transmitted ultrasound energy was demonstrated *in silico* and Tx-Rx parallel tracking (Fig. 2b) was designed to compensate for the transmit energy inhomogeneity. In experiment 1, neither qualitative nor quantitative differences were noted between Rx parallel tracking with different transmit F-numbers regarding HMI contrast and CNR. In experiment 2, Rx parallel tracking was compared with Tx-Rx parallel tracking in phantoms (Figs. 7 and 8) with smaller inclusion sizes, where still no statistically significant difference was found. In addition, since only one transmit-receive event is needed in Rx parallel tracking, it required less memory for data storage, transfer, and post-processing compared with Tx-Rx parallel tracking and PWC imaging. Therefore, Rx parallel tracking with focused transmits was concluded as the most robust and efficient among PW, PWC imaging, and Tx-Rx parallel tracking in on-axis displacement estimation, as demonstrated with HMI. Successful preclinical applications of Rx parallel tracking (Tx F/2.6) in HMI were shown in *ex vivo* human cancerous breast tissue with IDC (n = 1, Fig. 9) and *in vivo* murine models of triple-negative breast cancer (n = 2, Fig. 10).

A consistent 3° difference between consecutive compounding angles was used for PWC sequences in this study (3, 5, 7 angles *in silico* and 3, 5 angles in phantoms). In [50], [69], the authors have shown that the range of compounding angles in PWC determines the lateral resolution, which correlates with a transmit F-number as if focused transmits were used, while the angle difference between consecutive compounding frames defines the grating lobe locations. Accentuated grating lobes would present with a significant angle difference, and instead, trivial improvement in lateral resolution would be achieved with a slight angle difference in PWC. In this study, the effect of lateral resolution varied with the Tx F/# in parallel tracking, and the range of compounding angles in PWC was sought. Grating lobes between different PWC sequences were maintained unchanged by fixing the angle difference between consecutive PW frames. In phantom experiments, due to the limited time window for the pulse-echo tracking sequence (Fig. 1g), a maximum of 5 angles could be used in PWC without leading to interference between consecutive low-resolution PW frames. However, this was not a limitation *in silico*, where a maximum of 7 angles in PWC was simulated in Field II. Moreover, to implement the 5-angle PWC sequence in phantoms, the time delay between FUS excitation and speckle tracking was reduced from 90 to 40 μs. This potentially challenged the notch filtering of the FUS interference during channel data post-processing without affecting the imaging bandwidth, since this idle time was insufficient for the residual FUS interference to moderately attenuate prior to the pulse-echo tracking event. As a result, there were a few outliers inside the inclusion in the HMI map obtained by the 5-angle PWC sequence in Fig. 4. Preliminary work done by Li *et al.* [70] has shown promising results by using machine-learning-assisted filtering of the FUS interference in HMI; however, it needs further investigation to optimize the model.

For parallel tracking, a minimum of F/2.6 was used for the Tx F/#. This was the smallest F-number that a P12–5 transducer could achieve by using the full aperture (11.81 mm) to focus at 31 mm, where the focus of the imaging beam was co-aligned with the FUS focus. A maximum Tx F/# of 5 was selected for parallel tracking; this gave a beamwidth of around 1.2 mm, which was the lateral range for single scanning point displacement estimation. Since dynamic focusing was always implemented in receive, speckle biasing in receive was not expected, and therefore a constant Rx F/# of 2.6 was used across different transmit sequences.

Palmeri *et al*. first proposed the framework in [29], [57] for the simulation of ARFI imaging with focused imaging for speckle tracking using LS-DYNA3D and Field II. Later, Dahl *et al.* in [54] introduced Rx parallel tracking in ARFI imaging and showed promising results. Recently, Hossain *et al.* developed simulation work [56] for single-transducer HMI (ST-HMI) [37] with focused imaging. This paper demonstrated the first simulation work of PW and PWC imaging as well as focused imaging with Rx parallel tracking in ultrasound elasticity imaging. The FEM model was symmetric along the elevation direction. A preliminary comparison between FEM results without symmetry applied and with half symmetry was conducted in the inclusion and background, where no noticeable difference was found. Therefore, half symmetry [57] was applied to reduce computational overhead and single precision was used to minimize incurred computation memory. 30 dB Gaussian noise bandpass-filtered to the imaging bandwidth was added to the simulated channel signal to match the measured experimental channel SNR [60]. However, the effect of FUS interference on speckle tracking was not reproducible *in silico* due to model limitations.

The effect of speckle biasing in Rx parallel tracking for on-axis ultrasonic displacement estimation seen in Fig. 6 may be exaggerated *in silico* compared with HMI. First, it was challenging to fully simulate a similar inertia effect [30] as in phantoms and biological tissues with the FEM model. Considering a stronger inertia effect on-axis, the true displacement profile would be wider laterally. Another factor was the bandpass filtering process during the HMI displacement estimation based on the implemented AM frequency. This is one of the main advantages of HMI, where various noise sources and in-plane displacement artifacts can be corrected for by the distinct AM frequency. Therefore, even though a strong speckle biasing effect was observed in Fig. 6, no statistically significant difference was found between Rx and Tx-Rx parallel tracking in the phantom experiments. Meanwhile, Rx parallel tracking was more efficient and straightforward to implement compared to Tx-Rx parallel tracking. Thus, the preclinical demonstration of Rx parallel tracking with a transmit F-number of 2.6 in HMI was done in *ex vivo* IDC human cancerous breast tissues and two *in vivo* 4T1 murine tumors. The delineation of tumor boundaries and the differentiation of tumors between surrounding tissues were promising both *ex vivo* and *in vivo*. Future work involves applications of the optimized tracking beam sequence in *in vivo* human subjects.

A multi-element FUS transducer was used in this study for HMI synchronized with an imaging array controlled by a single 256-channel Vantage research system [49]. This eliminated the need for an arbitrary function generator to control the FUS transducer and another multi-channel ultrasound DAQ system to control the imaging array. Meanwhile, this setup had the potential to enable electronic beam steering of the FUS to reduce HMI data acquisition time [49], [53], [71], which is of critical benefit in clinical settings. Focused imaging with parallel tracking, however, is more complicated to implement with electronically beam-steered FUS than PW and PWC imaging, as the focused transmit beams of imaging must also be steered accordingly. While in PW imaging, the pressure field of the transmitted ultrasound wave already covers the entire field of view. Simultaneous beam steering of both the FUS excitation and focused imaging with parallel tracking will be implemented in the *in vivo* human study in the next step by co-registering the electronically steered beams of FUS and imaging with hydrophone measurement or displacement imaging.

As mentioned previously, one of the limitations of the P12–5 imaging array was that a minimum F-number of 2.6 could be used. A larger aperture, such as with an L7–4 or L14–22 linear array, could result in a smaller F-number to improve the lateral resolution further; however, a linear array is usually wider than the central opening of an annular FUS transducer so it would be challenging to co-align the two transducers. Although the P4–2 phased array has a slightly larger aperture, its lower center frequency could result in a higher displacement estimation jitter from ultrasonic speckle tracking based on the Cramér-Rao Lower Bound [65]. In fact, it is ideal to separate as much as possible the operating frequency bands and their harmonics of the imaging array and the FUS transducer. The leverage between these factors rendered the P12–5 phased array a good candidate. As demonstrated in Fig. 6 and in [54], a smaller transmit F-number could introduce more speckle biasing in parallel tracking. It is still interesting to study the effect of speckle biasing in Rx parallel with a smaller transmit F-number in HMI. Lastly, for the characterization of small lesions (< 2 mm) in HMI, instead of seeking to use a higher AM frequency, FUS with a smaller focal spot can also be exploited. To use such a system most effectively, focused imaging with a small F-number and high center frequency will be advantageous. Meanwhile, the 2D raster scanning grid format should also be optimized based on the FUS focal spot size.

Another interesting observation in Fig. 7 was that for small inclusions (2 mm), a higher AM frequency (800 Hz) produced higher contrast (Fig. 8a) partially because of a shorter wavelength of the generated mechanical wave, which complied with the study performed by our group [56]. A lower AM frequency (300 Hz) exhibited lower resolution in the axial direction, leading to an elongated inclusion shape in the displacement map, artificially showing the round inclusion as oval. On the other hand, a higher AM frequency is more prone to noise in homogeneous regions both inside the inclusion and in the background, which resulted in a lower SNR, in agreement with [72]. Therefore, the contrast was only comparable to that with a lower AM frequency in Fig. 8b. Note that the same frame rate (2000 Hz) for speckle tracking was used for different AM frequencies, which both satisfied the Nyquist sampling theorem. To further study the effect of AM frequency in HMI, the factor of samples per AM cycle must be ruled out by increasing the frame rates with the AM frequency and vice versa. Interestingly, larger inclusions even with the same Young’s modulus showed higher contrast and CNR in HMI maps at the same AM frequency. For AM frequencies above 1000 Hz, to capture the P2P displacement profile, the current pulse sequence needs to be adjusted to provide a frame rate of at least 2000 Hz to meet the Nyquist sampling theorem and to enhance displacement estimation robustness against noise for P2P displacement detection, a frame rate around 6000 to 8000 Hz is required [66]. PWC imaging improves the spatial resolution and contrast at the cost of reducing the frame rate by a factor of the number of compounding angles implemented. Even a 3-angle PWC sequence in this study could not achieve such a high frame rate, let alone for a 5-angle PWC sequence. Instead, Rx parallel tracking with focused transmits was able to produce a 9000 Hz frame rate. In general, higher AM frequencies were shown to be more favorable to contrast smaller inclusions in HMI [56] and lower AM frequencies were more beneficial for larger inclusions due to the reduced CNR with higher AM frequencies. *In vivo* study of multi-AM HMI sequences will be investigated in future studies with parallel tracking and steering.

Increased frame rates achieved with Rx parallel tracking not only help to detect tissue displacements at a higher AM frequency but also possibly improve the precision of phase shift estimation between the applied AM-ARF and the cumulative particle displacement for viscoelastic property measurement in quantitative HMI [73]. Vappou *et al.* [73] has shown that the HMI phase shift estimation on subjects with a relatively low viscosity was challenging, and increased frame rates could potentially increase the sensitivity of phase shift detection.

With the benefit of mechanical movement using the 3D positioner, a wide field of view covering over 35 mm laterally was produced for the visualization of the entire *ex vivo* human cancerous breast tissue including sufficient surrounding healthy tissue for analysis. DRs of lesion to the surrounding tissue were measured in both *ex vivo* human and *in vivo* murine cancerous breast tissues, which can provide important information for tumor characterization and tumor response assessment to treatments. Note that around 31 mm axially inside the murine tumors (Fig. 10), punctate hyperechoic structures were observed associated with low HMI displacements, i.e., high stiffness. Future studies will entail histological analysis for the characterization of such structures. Interestingly as expected, for a larger tumor in Fig. 10b, the DR of 0.12 was significantly lower than the DR of 0.32 measured in a smaller tumor in Fig. 10d. This indicates that malignant breast tumors stiffen during disease progression as reported previously [35].

The feasibility of implementing focused imaging with parallel tracking for displacement estimation from RF speckle tracking in HMI was demonstrated *in silico* and in tissue-mimicking phantoms with embedded elastic inclusions. The effect of speckle biasing in parallel tracking was also investigated and concluded as insignificant in HMI. Compared with PW and PWC imaging, improved contrast and CNR were achieved with parallel tracking. The optimized Rx parallel tracking sequence with a transmit F-number of 2.6 and 2D raster scanning with mechanical movement by a 3D positioning system were tested in an *ex vivo* human invasive ductal carcinoma (IDC) and *in vivo* murine models of breast cancer (4T1). Improved image quality indicated that HMI with Rx parallel tracking was promising for clinical studies. Other potential applications with Rx parallel tracking in HMI were also discussed.

## V. Conclusion

In this study, focused parallel tracking for HMI was implemented *in silico* and experimentally in an elastic phantom with various inclusion configurations. Consistent results were observed across simulations and phantoms. The effect of speckle biasing associated with parallel tracking was found to be negligible in the phantom experiments. Rx parallel tracking was concluded as more robust compared to plane wave imaging in terms of contrast and CNR, and more efficient than Tx-Rx parallel tracking and compounded plane wave imaging with comparable or slightly better HMI image quality. The feasibility of this new HMI tracking beam sequence was also demonstrated in *ex vivo* human cancerous breast tissues with an invasive ductal carcinoma and *in vivo* murine tumors of triple-negative breast cancer. Further clinical validations are suggested to have the proposed HMI sequence applied in tumor response assessment to chemotherapy and/or immunotherapy.
